# A sneak preview

**DOI:** 10.1007/s12471-021-01624-0

**Published:** 2021-09-09

**Authors:** A. W. G. J. Oomen

**Affiliations:** Department of Cardiology, Diakonessenhuis, Utrecht, The Netherlands

## Answer

**Fig. 1:**
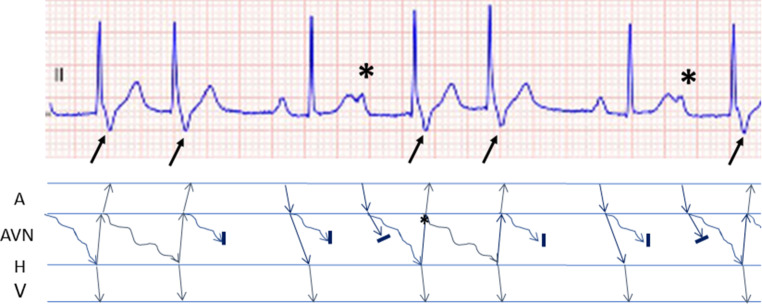
Ladder diagram showing the presumed mechanism of the arrhythmia. *A* atrium, *AVN* atrioventricular node, *H* His bundle, *V* ventricle

The ECG (Fig. 1 in the question) shows an irregular narrow QRS complex rhythm at a rate of approximately 100 beats/min. Some QRS complexes are preceded by P waves with a normal PQ time. They represent a normally conducted sinus rhythm. These complexes are followed by atrial extrasystoles hidden in the T waves (marked with asterisks in Fig. 1). The atrial extrasystoles are conducted with a prolonged PQ time. They are probably conducted via a slow pathway, while the fast pathway is still refractory from conducting the previous impulse. The next QRS complexes have a negative terminal deflection, suggesting a retrograde P wave (marked with an arrow in Fig. 1). Following the antegrade conduction via the slow pathway, the impulse is conducted retrogradely back to the atria via the fast pathway, causing so-called echo beats. This is depicted in a ladder diagram in Fig. 1. It is the typical start of AV nodal re-entrant tachycardia (AVNRT), although in this ECG, only two subsequent echo beats can be seen and no sustained tachycardia.

**Fig. 2:**
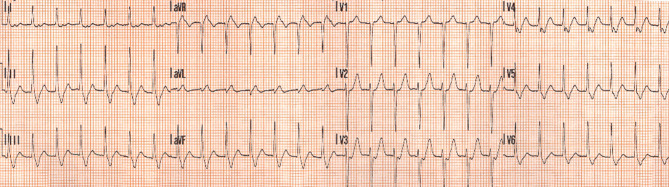
Electrocardiogram at presentation at the emergency department showing sustained supraventricular tachycardia very likely to be typical AV nodal re-entrant tachycardia

Two weeks later, the patient presented to the emergency department with palpitations. This time, the ECG rhythm was very likely to be that of typical AVNRT (Fig. 2). The patient was referred for electrophysiology. She was diagnosed with typical AVNRT, for which she underwent a slow pathway ablation. Since the ablation, she has been arrhythmia free.

A treatment dilemma would have arisen if the patient would not have presented with sustained arrhythmia. In that case, it would still have been reasonable to offer an electrophysiology study given the high likelihood of inducible sustained AVNRT.

